# Deep learning–based molecular morphometrics for kidney biopsies

**DOI:** 10.1172/jci.insight.144779

**Published:** 2021-04-08

**Authors:** Marina Zimmermann, Martin Klaus, Milagros N. Wong, Ann-Katrin Thebille, Lukas Gernhold, Christoph Kuppe, Maurice Halder, Jennifer Kranz, Nicola Wanner, Fabian Braun, Sonia Wulf, Thorsten Wiech, Ulf Panzer, Christian F. Krebs, Elion Hoxha, Rafael Kramann, Tobias B. Huber, Stefan Bonn, Victor G. Puelles

**Affiliations:** 1 III. Department of Medicine, University Medical Center Hamburg-Eppendorf, Hamburg, Germany.; 2 Institute of Medical Systems Biology, Center for Biomedical AI (bAIome), Center for Molecular Neurobiology (ZMNH), University Medical Center Hamburg-Eppendorf, Hamburg, Germany.; 3 Department of Nephrology and Clinical Immunology and; 4 Institute of Experimental Medicine and Systems Biology, RWTH Aachen University, Aachen, Germany.; 5 St.-Antonius Hospital Eschweiler, Department of Urology, Eschweiler, Germany.; 6 Department of Urology and Kidney Transplantation, Martin-Luther-University, Halle, Germany.; 7 Department of Pathology, University Medical Center Hamburg-Eppendorf, Hamburg, Germany.; 8 III. Department of Medicine, Division of Translational Immunology, and; 9 Hamburg Center for Translational Immunology, University Medical Center Hamburg-Eppendorf, Hamburg, Germany.; 10 Department of Internal Medicine, Nephrology and Transplantation, Erasmus Medical Center, Rotterdam, The Netherlands.

**Keywords:** Nephrology, Bioinformatics, Molecular pathology

## Abstract

Morphologic examination of tissue biopsies is essential for histopathological diagnosis. However, accurate and scalable cellular quantification in human samples remains challenging. Here, we present a deep learning–based approach for antigen-specific cellular morphometrics in human kidney biopsies, which combines indirect immunofluorescence imaging with U-Net–based architectures for image-to-image translation and dual segmentation tasks, achieving human-level accuracy. In the kidney, podocyte loss represents a hallmark of glomerular injury and can be estimated in diagnostic biopsies. Thus, we profiled over 27,000 podocytes from 110 human samples, including patients with antineutrophil cytoplasmic antibody–associated glomerulonephritis (ANCA-GN), an immune-mediated disease with aggressive glomerular damage and irreversible loss of kidney function. We identified previously unknown morphometric signatures of podocyte depletion in patients with ANCA-GN, which allowed patient classification and, in combination with routine clinical tools, showed potential for risk stratification. Our approach enables robust and scalable molecular morphometric analysis of human tissues, yielding deeper biological insights into the human kidney pathophysiology.

## Introduction

The kidney continuously filters blood and maintains overall body homeostasis, relying on a delicate balance between a complex vascular network and multiple specialized cell types (1). Podocytes are kidney epithelial cells with limited capacity for regeneration that function as master regulators of glomerular health (2). Experimental models show that severe podocyte loss leads to an irreversible process of progressive scarring, rendering the affected glomeruli nonfunctional (3–5). Furthermore, human podocyte loss has been identified in association with all major diseases contributing to chronic kidney disease (6–13).

Antineutrophil cytoplasmic antibody–associated glomerulonephritis (ANCA-GN) is primarily a systemic vasculitis with a strong immune-mediated epithelial reaction in the kidney, which leads to the formation of destructive glomerular lesions and a rapid loss of kidney function (14). While ANCA-GN has well-defined cellular changes (15) that include podocyte injury (16), podocyte loss is yet to be characterized in ANCA-GN patients. Using indirect immunofluorescence imaging, it is now possible to visualize different podocyte structures, facilitating the unambiguous identification of podocytes and, thereby, the quantification of podocyte depletion (5, 6). However, reliable image segmentation for routine clinical analysis remains challenging, mostly due to time constraints for detailed quantitative analysis with cellular resolution and lack of accuracy in available automated methods.

Time constraints, precision, and reproducibility are known hurdles in histopathology. For this reason, the automation of classification and quantification processes has the potential to lessen the diagnostic burden and improve the quality of the acquired data. Deep learning is increasingly gaining attention in multiple biomedical areas due to its potential clinical applications (17), including natural language processing (e.g., analysis of electronic health records) and computer vision (e.g., histopathology and radiology). Frameworks based on U-Net (a convolutional neural network specifically designed for the segmentation of images) are particularly interesting for histopathology (18, 19), since they can be used for image segmentation and specific tasks such as image-to-image translation (20, 21). To date, multiple reports have shown the high performance of deep learning networks for tissue-based classification of human disease (22, 23). Nonetheless, their role in detailed cellular morphometric profiling of clinical tissues remains unclear.

In this study, we present a deep learning–based workflow to perform cell-specific morphometric profiling of human kidney biopsies, including numbers, sizes, densities, and distributions of podocytes within their respective glomerulus, which allowed a comprehensive characterization of endpoint variability within and between patients. We analyzed a total of 1095 glomeruli from 110 patients to profile 27,696 podocytes based on tissue expression of 2 complementary antigens in order to identify, segment, and quantify podocyte depletion. A previously unrecognized morphometric signature of podocyte depletion was detected in patients with ANCA-GN (as listed in Supplemental Table 1; supplemental material available online with this article; https://doi.org/10.1172/jci.insight.144779DS1), allowing patient classification with near–human level accuracy and showing potential for risk stratification when combined with established clinical tools. Our findings suggest that focal podocyte loss may be a transitional state before the onset of overt lesion formation in patients with ANCA-GN. Together, these findings highlight the potential of deep learning–based architectures for enabling robust and scalable molecular morphometric analyses of human tissues.

## Results

### Morphometric profiling of human samples using a dual segmentation U-Net.

**Figure 1:**
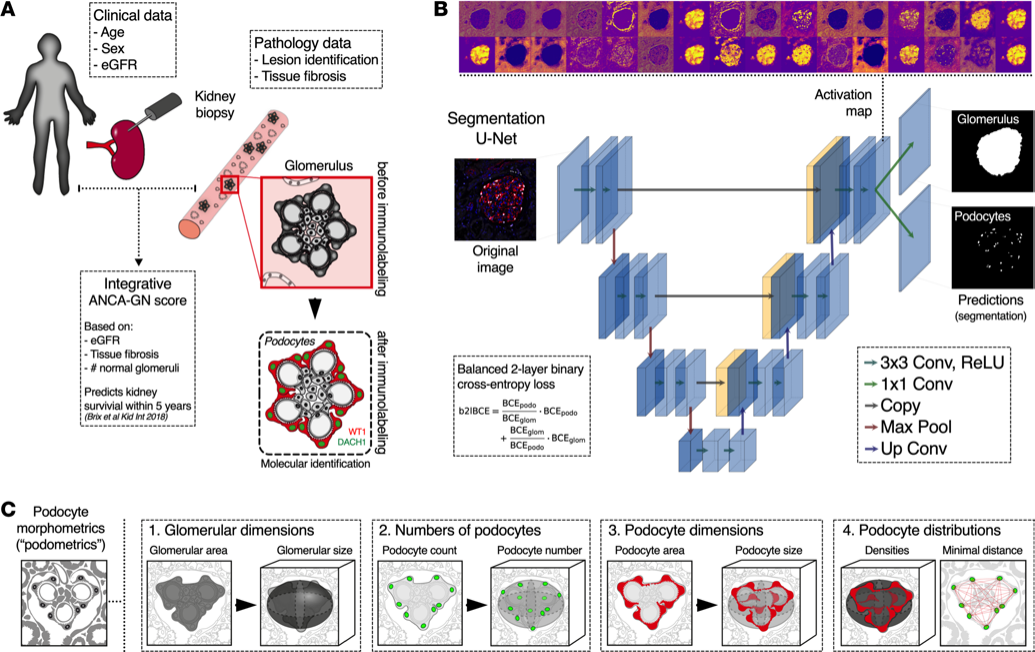
Segmentation U-Net for molecular morphometrics in kidney samples. (**A**) Biopsies from patients with immune-mediated kidney diseases, which are diagnosed, treated, and monitored based on clinical, pathological, and integrative data, are used to perform molecular labeling of kidney podocytes, based on indirect immunofluorescence (24). (**B**) Glomerular area and podocyte nuclei are virtually dissected from high-resolution confocal images with a segmentation U-Net for 2 simultaneous outputs that was trained using a balanced 2-layer binary cross-entropy loss. (**C**) 3D podocyte morphometrics (podometrics) were generated by model-based stereology, which extrapolates 3D from 2D data; in this case, glomerular and podocyte areas and podocyte spatial location were used to estimate 3D glomerular dimensions, as well as numbers, sizes, and distributions of podocytes. ANCA-GN, antineutrophil cytoplasmic antibody–associated glomerulonephritis; eGFR, estimated glomerular filtration rate; DACH1, Dachshund Family Transcription Factor 1; WT1, Wilms’ Tumor 1; BCE, binary cross-entropy; Conv, convolution; ReLU, rectified linear unit; Max Pool, max pooling.

Human kidney biopsies from patients with available clinical data (i.e., age, sex, and estimated glomerular filtration rate [eGFR]), pathological endpoints (i.e., interstitial fibrosis), and integrative scores (i.e., ANCA-GN score) were immunolabeled using antibodies against podocyte-specific transcription factors, including nuclear expression of Dachshund Family Transcription Factor 1 (DACH1) and cytoplasmic expression of Wilms’ Tumor 1 (WT1), in order to unambiguously identify glomerular podocytes (Figure 1A) and carefully profile a total of 27,696 podocytes. A total of 1095 immunolabeled images was used for training, validation, and testing during the development of the deep learning architectures, including 722 images from 48 controls and 373 images from 62 patients with ANCA-GN. General patient demographics are outlined in Supplemental Figure 1.

Common definitions of machine learning–related language are provided in Table 1. In general, deep learning architectures for image analysis consist of convolutional layers that analyze the image sequentially in order to extract increasingly complex features. During this process, the image size is conventionally reduced through max-pooling (down-sampling), encoding the data in a smaller dimension. To obtain segmentation predictions of the same size as the input image, a decoder restores the information to the original size via upconvolutions combined with additional convolutional layers. Following this logic, we developed a dual output deep learning–based segmentation architecture (U-Net) that has an encoder/decoder structure with 3 convolutional layers, each containing between 32 (first/last layers) and 256 filters (bottom layer), which can simultaneously extract glomerular and podocyte nuclear areas from a composite fluorescence image (Figure 1B). Segmented areas are integrated into model-based stereology formulas that estimate podocyte morphometrics (podometrics), including glomerular dimensions, numbers of podocytes, and podocyte dimensions and distributions (i.e., minimal distances between neighboring podocytes) within each glomerulus (Figure 1C).

The parameters or weights of the U-Net convolutional layers are iteratively updated in a training process, which consists of multiple epochs (or temporal steps). In each epoch, all training images are passed through the network once. To update the weights of the network, the difference between the network’s prediction and the manually annotated ground truth is determined for each image based on a “loss function.” We used a balanced 2-layer binary cross-entropy loss function that adaptively accounts for the performance of each individual segmentation task (Supplemental Figure 2, B–D). Once the images of the validation set are passed through the network, predictions are computed and evaluated. This way, the performance of the network is monitored during training, and the best weights and hyperparameters can be determined. The held-out test set is only used to evaluate the best network, which thus provides unbiased results. In this study, the overall quality of the segmentation performed by the U-Net has been evaluated using standardized metrics that measure the similarity between prediction and ground truth of the glomerular and podocyte segmentations at pixel and object levels (i.e., Dice scores, where a Dice score of 0 indicates no overlap of prediction and ground truth and 1 a perfect match).

In order to ensure an optimal performance of our U-Net, hyperparameters were determined using cross-validation, where we confirmed that the number of training images was sufficient to achieve Dice scores over 0.90 (Supplemental Figure 2A). We compared our dual-output segmentation U-Net to 2 single-output U-Nets (for glomerular and podocyte nuclear areas separately), showing similar results (Supplemental Figure 3, A and B), which means that we can work with fewer parameters and require less training and evaluation time for the dual-output U-Net than for the 2 single-output U-Nets. Furthermore, our dual-output U-Net outperformed a customized ImageJ-based segmentation script at pixel and object levels, with a strong reduction in false-positive rates (Supplemental Figure 4, A and B).

### U-Net cycleGAN for annotation-free bias minimization.

A lack of generalization is a well-known vulnerability of deep learning architectures (17). To this end, we first compared podometrics obtained from the same patients who were systematically imaged in 2 different locations with different microscopes and by different operators with different levels of microscopy experience. Podocyte density was not affected by these different conditions (Supplemental Figure 5A), neither at a patient level nor at a glomerular level (Supplemental Figure 5B), when the segmentation U-Net was trained jointly on these data sets. However, we observed significant differences in the variance of DACH1 or WT1 expression per image (pixel level) (Supplemental Figure 5C), suggesting that batch effects and image bias should be addressed in order to increase the reproducibility and scalability of the method.

**Figure 2:**
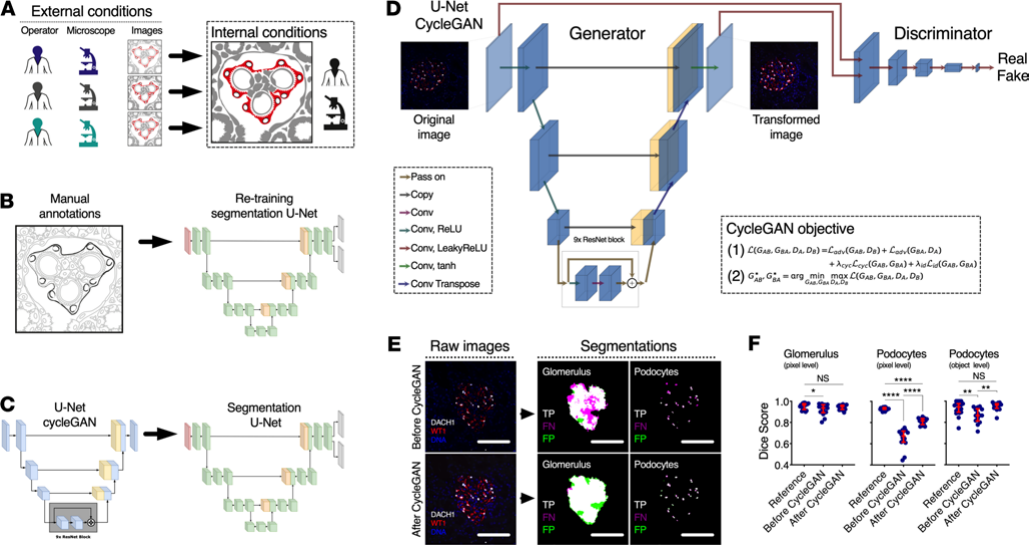
U-Net cycleGAN for bias minimization between different data set domains. (**A**) Scalable frameworks require adaptability to external conditions. While indirect immunofluorescence protocols can be standardized, operator training, microscopy set-up, and eventually image quality are hard to control, especially if segmentation tasks have been defined based on tightly controlled internal conditions. (**B**) The images can be annotated manually in order to retrain the segmentation U-Net before it is applied to a new data set. (**C**) Alternatively, we propose using a U-Net cycleGAN (without annotations) in order to transform images before applying the segmentation U-Net. (**D**) While the generator in the U-Net cycleGAN transforms images from one data set domain to the other, the discriminator tries to distinguish between “real” and “fake” images. This adversarial game is reflected in the cycleGAN objective, which is made up of the adversarial loss L_adv_, the cycle-consistency loss L_cyc_, and an identity loss L_id_. (**E**) Representative images showing segmentation agreement with ground truth and reductions in false negatives. (**F**) Dice score at both pixel and object level significantly improved after cycleGAN for podocytes (*n* = 20 images for the reference and *n* = 24 images for before/after U-Net cycleGAN; Kruskal-Wallis with Dunn’s multiple-comparisons tests were performed). In dot plots, every blue dot represents 1 image, and red error bars represent medians and IQRs. Conv, convolution; ReLU, rectified linear unit; tanh, hyperbolic tangent; DACH1, Dachshund Family Transcription Factor 1; WT1, Wilms’ Tumor 1; TP, true positives; FP, false positives; FN, false negatives. ****P < 0.0001, **P < 0.01, *P < 0.05. Scale bars: 150 μm.

Multiple operators and microscopes led to differences in image quality, differing from the reference data set (Figure 2A). One solution is to continuously retrain the segmentation U-Net (Figure 2B), which progressively leads to a more robust network but requires manual annotations. An alternative approach can be found in the use of deep learning–based annotation-free bias minimization (Figure 2C). Thus, we implemented a U-Net cycleGAN (cycle-consistent generative adversarial network with a U-Net–like generator) to transform images obtained under different conditions (i.e., microscope and operator) into images resembling the reference data set used for training the segmentation network (Figure 2D). Representative images show the resulting segmentation optimization (Figure 2E) and improvements in Dice scores at both pixel and object levels (Figure 2F). Training curves of the U-Net cycleGAN, as well as receiver operating characteristic (ROC) and precision-recall curves for the different combinations of data and segmentation networks, are provided in Supplemental Figure 6, A and B. While these results provide evidence that unannotated data sets can be efficiently segmented using a network trained on the reference data set when they are bias transferred using the U-Net cycleGAN (e.g., improvement of the mean podocyte pixel–based Dice score from 0.65 to 0.81; see center and right panel of Supplemental Figure 6C), we obtained slightly better segmentation results using a segmentation U-Net trained on all images, including the 2 control and the ANCA-GN data sets (mean podocyte pixel-based Dice score, 0.84; left panel of Supplemental Figure 6C). All further results are, therefore, based on the U-Net trained jointly on the 2 control and the ANCA-GN data sets.

### Molecular podometrics reveal podocyte loss in ANCA-GN.

**Figure 3:**
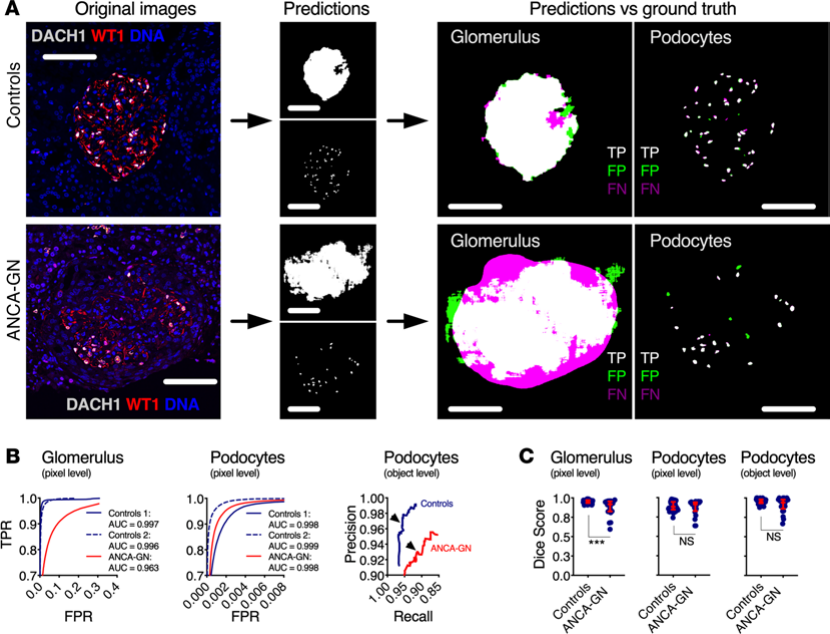
Application of segmentation U-Net to human kidney biopsies. (**A**) Visual representation of the segmentation process, from original images, to segmentation outputs for glomeruli and podocytes, and their respective correlation with manually segmented ground truths, highlighting true positives, false positives, and false negatives. (**B**) Receiver operating characteristic (ROC) and precision-recall curves in samples from controls and ANCA-GN patients; arrowheads show selected thresholds for both conditions (*n* = 20 images for Controls 1, *n* = 24 images for Controls 2, and *n* = 21 images for ANCA-GN patients). (**C**) Dice scores at pixel and object levels for glomeruli and podocytes, showing comparable segmentation performance in health and disease (*n* = 44 images for controls and *n* = 21 images for ANCA-GN patients; Mann-Whitney *U* tests were performed). In dot plots, each blue dot represents 1 image, and red error bars represent medians and IQRs. ANCA-GN, antineutrophil cytoplasmic antibody–associated glomerulonephritis; DACH1, Dachshund Family Transcription Factor 1; WT1, Wilms’ Tumor 1; TPR, true positive rate; FPR, false positive rate; TP, true positives; FP, false positives; FN, false negatives. ***P < 0.001. Scale bars: 100 μm.

Representative images show high accuracy and precision of the U-Net for podocyte segmentation in samples from both controls and ANCA-GN patients (Figure 3A); this was illustrated by ROC and precision-recall curves (Figure 3B). Strong agreement between ground truth and U-Net outputs was determined by pixel- and object-based Dice scores (mean podocyte pixel- and object-based Dice scores for controls 0.86 and 0.95, respectively) (Figure 3C). While image segmentation in ANCA-GN patients was comparable with controls, detection levels were not identical (mean podocyte Dice scores for ANCA-GN patients 0.87 and 0.91, respectively). For this reason, we compared segmented areas from glomeruli and podocytes in the ground truth and those obtained from the U-Net, which showed identical differences between controls and ANCA-GN patients (Supplemental Figure 7, A and B); this supports biological differences rather than technical artifacts. Furthermore, we also determined direct correlations between ground truth and U-Net segmentation outputs from both controls and ANCA-GN patients (Supplemental Figure 7C).

**Figure 4:**
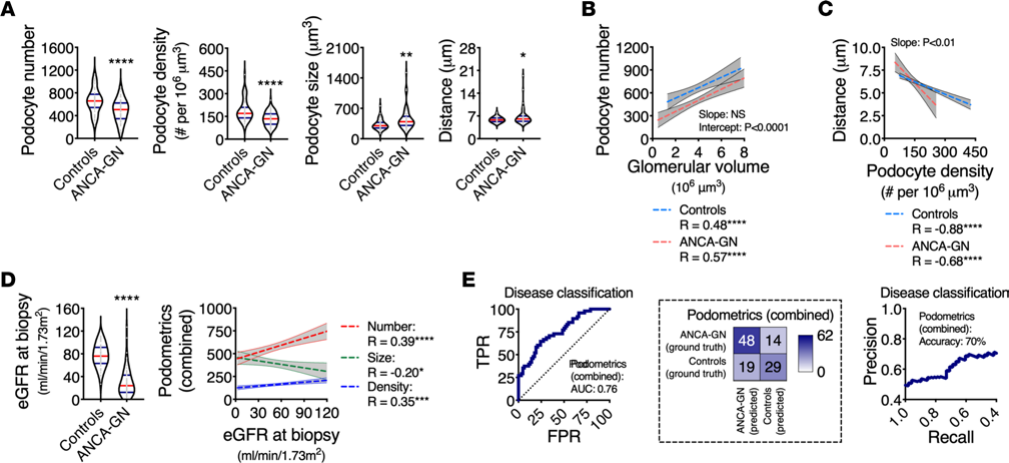
Molecular podometrics reveal podocyte loss in patients with ANCA-GN. (**A**) Podocyte morphometric analysis (podometrics; median per patient) showing reductions in podocyte numbers and densities, as well as increases in podocyte sizes and closest neighbor distances in ANCA-GN patients compared with controls. (**B**) Spearman’s rank correlation analyses confirm a pattern of podocyte loss across the entire range of glomerular volume. (**C**) Increases in podocyte closest neighbor distances are associated with reductions in podocyte density. (**D**) ANCA-GN patients have a lower estimated glomerular filtration rate (eGFR) at the time of biopsy compared with controls; features of podocyte depletion are associated with eGFR at biopsy. (**E**) Receiver operating characteristic (ROC) and precision-recall curves of a logistic regression using leave-one-out cross-validation showing the discrimination power of combined podometrics (podocyte number, density, and size), including confusion matrix. In all panels *n* = 48 patients for controls and *n* = 62 patients for ANCA-GN; Mann-Whitney *U* tests were performed. In violin plots, each gray dot represents the median value per subject, red lines represent medians, and blue lines represent IQRs. Regression lines represent lines of best fit and 95% CI. ANCA-GN, antineutrophil cytoplasmic antibody–associated glomerulonephritis; eGFR, estimated glomerular filtration rate; TPR, true positive rate; FPR, false positive rate. ****P < 0.0001, **P < 0.01, and *P < 0.05.

Reductions in median podocyte numbers and densities with consequent increases in median podocyte sizes and distances between closest neighbors were found in patients with ANCA-GN compared with controls (Figure 4A). The median glomerular size was directly associated with median podocyte number (*R* = 0.48, *P* < 0.0001 in controls, and *R* = 0.57, *P* < 0.0001 in ANCA-GN) with significant differences in the intercept (*P* < 0.0001), which suggests podocyte loss across the entire spectrum of glomerular size (Figure 4B). Similarly, median podocyte density was inversely associated with median minimal distances between neighboring podocytes (*R* = –0.88, *P* < 0.0001 in controls, and *R* = 0.68, *P* < 0.0001 in ANCA-GN) with statistical differences in the slope (*P* < 0.01), suggesting that compensatory podocyte hypertrophy is exacerbated in ANCA-GN patients (Figure 4C).

In our cohort, the main clinical discriminator between controls and ANCA-GN patients was kidney function, assessed by eGFR at the time of biopsy. In particular, eGFR was associated with podocyte number (*R* = 0.39, *P* < 0.0001), density (*R* = 0.35, *P* < 0.001) and size (*R* = –0.20, *P* < 0.05) (Figure 4D). Using leave-one-out cross-validation, we generated a combined podometric score, including podocyte number, density, and size, which also partially discriminated between controls and ANCA-GN patients in a logistic regression with an AUC of 0.76 (Figure 4E). Together, these findings show a potential overlap in the levels of podocyte depletion between controls and ANCA-GN patients.

### Morphometric signature of podocyte depletion identifies patients with ANCA-GN.

Lesion development in ANCA-GN is focal, meaning that, within the same patient, some glomeruli are affected and others are not (Supplemental Figure 8A). This is directly reflected in the changes in the variances per subject of all podometric parameters (Supplemental Figure 8B), which decreased in podocyte numbers and densities, but increased in sizes and distances between closest neighbors.

**Figure 5:**
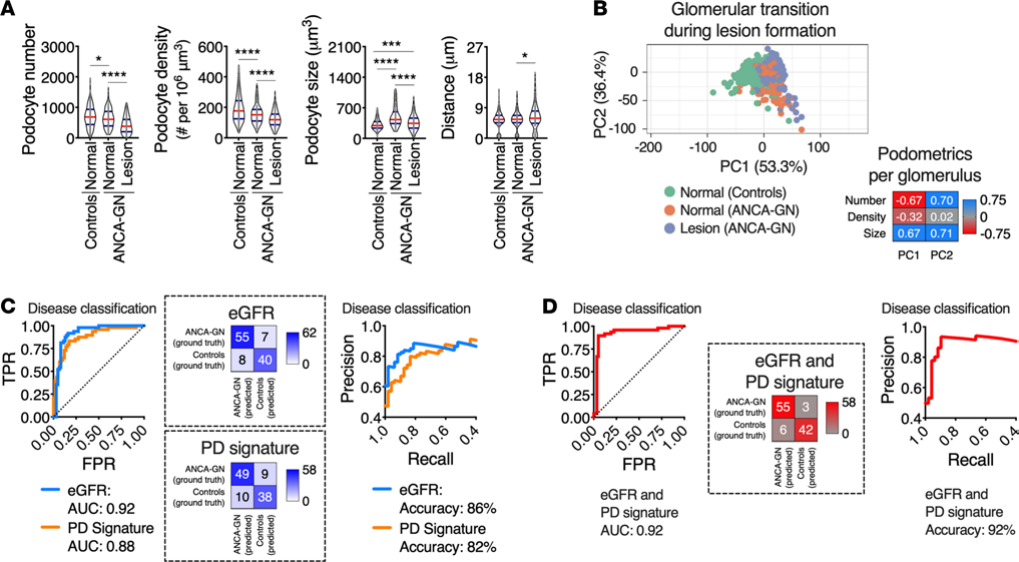
Podocyte morphometric signature identifies ANCA-GN patients. (**A**) Podocyte-morphometric analysis (podometrics; per glomerulus) showing a pattern of podocyte loss and hypertrophy in glomeruli classified as “normal” (without lesion) in ANCA-GN patients. (**B**) Principal component analysis (PCA) using Pareto scaling to rows. Probabilistic PCA was used to calculate principal components, confirming that normal glomeruli in ANCA-GN patients represent a transitional state between normal glomeruli in controls and lesions in ANCA-GN patients. In **A** and **B**, *n* = 722 normal glomeruli for controls and *n* = 373 glomeruli for ANCA-GN patients; Kruskal-Wallis with Dunn’s multiple-comparisons tests were performed. (**C**) Receiver operating characteristic (ROC), precision-recall curves, and confusion matrices of patient classification with a logistic regression using leave-one-out cross-validation based on eGFR and on a morphometric signature of podocyte depletion (PD), which combines morphometric data from every available glomerulus per biopsy per patient. (**D**) ROC, precision-recall curves, and confusion matrices for eGFR and PD signature as classifiers. In **C** and **D**, *n* = 48 patients for controls and *n* = 58 patients for ANCA-GN with PD signature; *n* = 62 patients for ANCA-GN with eGFR only. In violin plots, each gray dot represents 1 glomerulus, red lines represent medians, and blue lines represent IQRs. ANCA-GN, antineutrophil cytoplasmic antibody–associated glomerulonephritis; eGFR, estimated glomerular filtration rate; TPR, true positive rate; FPR, false positive rate. ****P < 0.0001, ***P < 0.001, and *P < 0.05.

Analyses of single glomeruli showed that podocyte depletion was present in ANCA-GN patients, even in glomeruli that were not defined as glomerular lesions, and was associated with compensatory podocyte hypertrophy (Figure 5A). A principal component analysis (PCA) also revealed that normal glomeruli in ANCA-GN patients represent a transitional state from normal glomeruli in controls to overt glomerular lesions in ANCA-GN (Figure 5B), suggesting that analyses of individual glomeruli within one patient may provide additional clues that may be applied to differentiate controls and ANCA-GN patients. Using leave-one-out cross-validation, we generated a morphometric signature of podocyte depletion, which is generated per subject based on all available morphometric data, including both central tendencies and measures of variability. Importantly, this integrative parameter discriminated between controls and ANCA-GN patients in a logistic regression, as shown in both ROC (Figure 5C) and precision-recall curves (AUC, 0.88) with an accuracy of 82% (Figure 5D), which was almost identical to the discrimination power of eGFR (AUC, 0.92; accuracy, 86%).

In this cohort, 3 ANCA-GN patients were classified as controls and 6 controls were classified as ANCA-GN. First, we hypothesized that this could be due to segmentation artifacts, since DACH1 expression is upregulated in other cell types (i.e., erythrocytes and proximal tubular cells). However, we carefully screened all images from these 9 misclassified subjects and confirmed appropriate segmentation; representative images are shown in Supplemental Figure 9A. In patients with ANCA-GN, misclassified subjects were younger and had higher eGFR than median values for controls. In controls, misclassified cases were older and had lower eGFR than median values for ANCA-GN patients (Supplemental Figure 9, B and C). Together, these findings suggest that misclassifications may be associated with early stages of disease in ANCA-GN and age-related podocyte loss in controls. Furthermore, this morphometric signature of podocyte depletion marks the degree of disease progression in close relation to physiological readouts.

### Potential of podometrics for risk stratification in patients with ANCA-GN.

**Figure 6:**
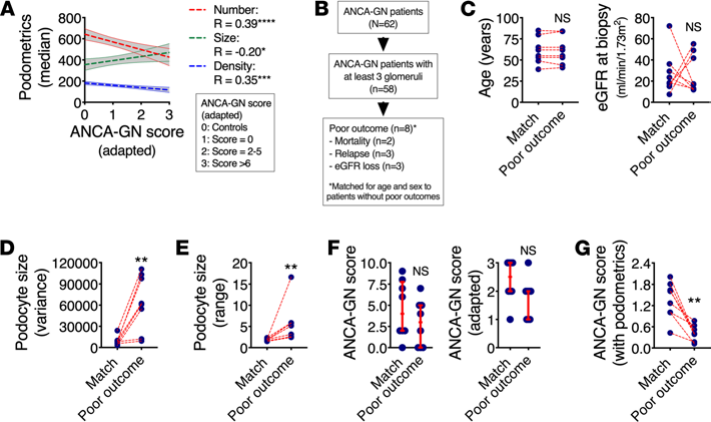
Potential role of podometrics for ANCA-GN risk evaluation. (**A**) Features of podocyte depletion correlate with an adapted ANCA-GN score that predicts poor clinical outcomes within 5 years (*n* = 62 patients for ANCA-GN; Spearman’s rank correlation analyses were performed). (**B**) Among all 62 ANCA-GN patients, clinical follow-up data identified a total of 8 patients with poor clinical outcomes, including mortality, relapse, and loss of estimated glomerular filtration rate (eGFR) of at least 15% from baseline; these were carefully age- and sex-matched to patients without negative outcomes. (**C**) Successful age-match with random selection of variable eGFR. (**D**) Variance in podocyte size per biopsy was significantly elevated in patients with poor outcomes. (**E**) The ratio between maximal and minimal podocytes sizes (range) per biopsy was also increased in patients with poor outcome. (**F**) Neither the classical ANCA-GN score nor an adapted ANCA-GN score were different between patients with poor outcome and matched controls. (**G**) A modified ANCA-GN score based on a ratio between the adapted ANCA-GN score and the range of podocyte size per biopsy was significantly reduced in patients with poor outcome. In **C**–**G**, *n* = 8 ANCA-GN patients with negative outcomes were carefully age- and sex-matched to *n* = 8 ANCA-GN patients without negative outcomes; Mann-Whitney *U* tests were performed. Regression lines represent lines of best fit and 95% CI. Each blue dot represents 1 subject. In **F**, red lines represent medians and IQRs. ANCA-GN, antineutrophil cytoplasmic antibody–associated glomerulonephritis. ****P < 0.0001, ***P < 0.001, **P < 0.01, and *P < 0.05.

A recent study proposed an integrative predictive score of 5-year kidney survival in ANCA-GN (24), based on eGFR, percentage of interstitial fibrosis, and number of nonpathological glomeruli. We adapted this ANCA score to include a baseline comparison with control patients and model associations to podometrics, showing that median podocyte number, density, and size are significantly correlated with the modified ANCA-GN score (Figure 6A). From a total of 62 patients with ANCA-GN, 58 had at least 3 identified glomeruli in the diagnostic biopsy, which allowed us to perform analysis of intrasubject variability. Then, 8 patients were identified based on our definition of “poor outcomes,” including mortality, relapse, or loss of at least 10% of eGFR within their respective follow-up period (Figure 6B). For a balanced comparison, we carefully matched these 8 subjects for age and sex within the remaining available patients from our cohort (*n* = 8 matched ANCA-GN patients). While our matching strategy was successful for age and sex, we were not able to obtain matches by eGFR (Figure 6C). Variances (Figure 6D) and ranges (Figure 6E) in podocyte size were significantly increased in ANCA-GN patients with poor outcomes. Neither the conventional ANCA-GN score nor our adapted version were different between the outcome groups (Figure 6F), but a ratio between the adapted ANCA-GN score and range in podocyte size showed significant differences by outcome group (Figure 6G). In summary, these findings highlight a potential for additional risk stratification among ANCA-GN patients using a combination of podometrics and available clinical and pathological tools.

## Discussion

In this study, we present a deep learning–based approach that automatically identifies morphometric signatures of podocyte depletion in human kidney biopsies, achieving human-level accuracy while saving time and resources. Our method provides robust and scalable molecular morphometric endpoints for patients with ANCA-GN, revealing potentially novel pathophysiological insights of kidney epithelial biology and serving as an example for the potential integration of deep learning–based technologies into clinical settings.

Previous deep learning studies focused on the end-to-end evaluation of biopsies through classification into several categories based on classical histology (22, 23, 25–27). To the best of our knowledge, this is the first report to combine deep learning for object segmentation in clinical samples with cell-specific morphometrics, which does not only allow disease classification and risk stratification, but also provides objective endpoints for the analysis of kidney biopsies. Furthermore, antigen-based cellular identification reduces subjectivity in annotation strategies, since extensive specialized training is not needed in order to identify protein expression with fluorescence microscopy, accelerating annotations and homogenizing ground truth definition — all of which are well-defined obstacles for clinical translation of deep learning–based methodologies (17). However, reproducibility remains a valid drawback for new clinical tools — especially those dependent on microscopy.

Bias minimization through a U-Net cycleGAN allows a wider use of the pipeline, given that data obtained by various users and on different microscopes can be adapted in order to efficiently homogenize image quality. Generative networks have been used in the past for histopathological analysis but mostly have been limited to classical histological stainings (28, 29). While this strategy is certainly effective and is comparable with multi–data set training, manual annotations and retraining of the segmentation architecture is the safest approach to maximize accuracy. In this manuscript, we provide both options, allowing users to decide based on their experimental and clinical needs.

The limited sample size for training, optimization, validation, and testing of multiple deep learning architectures may be perceived as a shortcoming of the present study. However, this is a very common problem in biomedical sciences. The number of patients with follow-up data and negative outcomes, even with extended internal criteria (i.e., at least 10% of eGFR), prevented us from providing predictive analyses at this stage. For this reason, our observations should be taken as proof-of-principle and will need careful validation in larger patient cohorts with longer and standardized follow-up periods. Furthermore, the successful integration of artificial intelligence–based morphometrics into clinical practice will not only depend on larger data sets, but also on standardization and automation of tissue processing and imaging. While our efforts for batch effect minimization are promising, we only tested variations in image quality based on 2 parameters: microscopy operators and confocal systems. The compatibility of our approach with other high-throughput imaging methodologies, such as spinning disk and epifluorescence–based systems, still needs to be validated.

The devastating nature of ANCA-GN requires continuous efforts to identify diagnostic and prognostic tools that may guide clinical management (15). In the pathophysiology of lesion formation during ANCA-GN development and progression, it is known that immune cells and parietal epithelial cells play key roles (1, 14, 16). Importantly, our data highlight a previously unrecognized role of podocyte loss in ANCA-GN that could only be revealed by analyzing single glomeruli and their variability within and between subjects. The unexpected value of podometric endpoints in diagnostic ANCA-GN biopsies can only strengthen the position of podocyte depletion as a hallmark of glomerular disease (30, 31). Future studies will assess whether podocyte depletion signatures may serve as objective endpoints for the management of glomerular diseases, as well as their potential applicability to patient diagnosis and prognosis. It is our hope that this study may pave the way for the development and implementation of advanced tissue morphometrics in routine clinical pathology.

## Methods

### Human samples.

Tissue collection from nephrectomy samples due to renal cell carcinoma was performed at Eschweiler Medical Center. After fixation with 4% paraformaldehyde (PFA), representative kidney blocks from the pole opposite to the tumor were extracted — a strategy that aimed to collect nonpathological tissue. Kidney biopsies from patients with ANCA-associated glomerulonephritis were obtained from the Hamburger Glomerulonephritis Registry (https://www.sfb1192.de/en/register).

### Immunofluorescence and confocal microscopy.

Previously reported protocols were applied (5, 6). To identify podocytes, we used a combination of WT1 (Agilent Technologies; IS05530-2) and DACH1 (Sigma-Aldrich; HPA012672) (32) as primary antibodies; Alexa-Fluor 488, -555, and/or -647 as secondary antibodies (Invitrogen; A21202, A31572, A31571, and A31573, respectively) depending on the experiment; and a DNA marker to identify single nuclei — either DAPI (Sigma-Aldrich; D9542) or DRAQ5 (Abcam; ab108410). Optical images were obtained using inverted laser confocal microscopes (Nikon and LSM800, Zeiss), stored in 1024 × 1024 pixel frames. Each image contained 1 glomerulus.

### Manual image annotation for ground truth generation.

Ground truth data sets were generated based on podocyte nuclei and glomerular areas in manual segmentation performed by 3 expert scientists trained under equal conditions within our team, blinded from the patient data. Quality control was performed by a senior scientist within our team. During training, the segmentation U-Net then learned from the annotated images (training and validation sets) to segment the structures of interest, and the final results were validated on another set of annotated images (test set).

Glomeruli were classified as normal or lesions based on anatomical criteria. Normal glomeruli had a monolayer of parietal epithelial cells and glomerular tufts with homogenous and robust podocyte labels, namely cytoplasmic WT1 and nuclear DACH1. Glomerular lesions showed at least a double layer of parietal epithelial cells, capillary collapse, and/or segmental or global absence of podocyte labeling.

### ImageJ baseline script for glomerulus and podocyte nuclei segmentation.

In order to segment the glomerulus using ImageJ, the following sequence was used: (a) channel splitting; (b) thresholding and then dilation applied to each channel separately; (c) channel merging; (d) filling holes, eroding, and particle analysis; and (e) selection of the biggest region of interest. In order to segment podocyte nuclei using ImageJ, the following sequence was used: (a) channel splitting and thresholding; (b) dilation of WT1 channel followed by combination of all channels using the logical operator “AND”; (c) thresholding of DNA label; (d) dilation, filling holes, and eroding; and (e) distance transformation using watershed (MorphoLibJ plugin).

### Dual-output segmentation U-Net.

Inspired by Ronneberger et al. (18, 19), a U-Net architecture was implemented in Python 3 using Tensorflow 1.13. The segmentation U-Net consists of an encoder with 3 layers, where the convolutions in the first layer have 32 filters. The number of filters is doubled in the following layers. After the bottom layer with 256 filters, the number of filters is halved again for each of the 3 layers of the decoder. We pad the images in order to receive segmentations of the same size as the input images.

The U-Net was modified to simultaneously return a dual segmentation output: glomerular areas and podocyte nuclear areas. An annotated subset of images (*n* = 317) was split into training (192 images), validation (60 images), and test (65 images) subsets with the relation of approximately 60/20/20, maintaining that all images from 1 subject should belong to 1 subset. For training, the use of extensive on-the-fly data augmentation (horizontal and vertical flips, horizontal and vertical shifts, rotations up to 45°) was important for the generalizability of the network. The network was trained for 2000 epochs with a batch size of 2 images on an Nvidia Tesla V100 graphics card. We used RMSprop as an optimizer and introduced a custom balanced 2-layer binary cross-entropy loss that adaptively takes into account the current performance of each segmentation task. The binary cross-entropy for each task,

 (Equation 1)
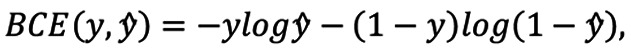


for mask (*y*) and prediction (*ŷ*) (the prediction is clipped to lie between ε and 1 – ε, with ε = 1 × 10^–7^, in order to avoid logarithms of 1 and, thus, later divisions by 0) is adapted to consider both segmentation tasks simultaneously and to weight each term so that the currently poorer performing task receives more importance:

(Equation 2)
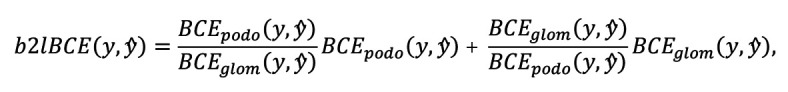


where *BCE_podo_* and *BCE_glom_* are the binary cross-entropies for the podocyte and glomerulus segmentation tasks, respectively. Additionally, we weighted foreground objects and the background in the training loss in order to enforce a better segmentation of narrowly spaced podocytes. This was done using the function weight maps *w*(*x*) for each image using its ground truth mask *x* similar to those proposed by Falk et al. (19):

 (Equation 3)
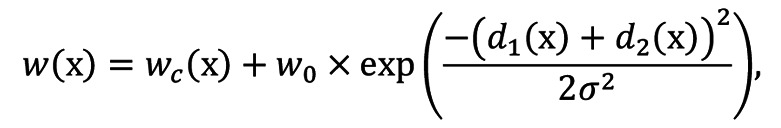


where *w_c_* is the class probability map for the mask, *d*_1_ is the distance to the border of the nearest cell, d_2_ is the distance to the border of the second nearest cell, *w*_0_ a coefficient that controls the importance of the distance maps, and σ^2^ the variance of the Gaussian filter.

Our evaluation metric is the commonly used Dice score, evaluated for each task separately at pixel level and additionally at object level for the podocytes. Incomplete nuclear parts or glomeruli were filtered out using postprocessing, removing all objects smaller than 800 μm for glomeruli and smaller than 3 μm for podocyte nuclei. Given that this architecture provided excellent results, and some tests with a more complex architecture (i.e., Mask R-CNN) yielded similar results, we decided to work with our more compact and faster dual U-Net.

### Hyperparameter optimization.

In order to find the optimal architecture and hyperparameters, an extensive grid search across various options was performed using Ray Tune (https://docs.ray.io/en/master/tune/index.html) and Sacred (https://sacred.readthedocs.io/en/stable/). Chosen values are in parentheses: single versus dual segmentation (dual), number of layers (*n* = 3), number of filters in the first layer (*n* = 32), dropout in encoder and decoder (no), dropout in the bottom layer (yes), skip connections between encoder and decoder (yes), dropout in the skip connections (no), batch normalization (yes), optimizer (RMSprop), learning rate (1 × 10^–5^), learning rate decay (no), loss (balanced 2-layer binary cross-entropy), weighting (yes), histogram equalization (no), contrast stretching (no), data augmentation (yes), and oversampling of crescents (no). To evaluate these, iteratively, a few (related) hyperparameters were varied. Then, using 4-fold cross-validation on the combined training and validation subsets of the Controls 1 data set (Supplemental Table 1), networks were trained, and the optimal configuration was chosen based on the average Dice scores, as well as their SD between the different folds of the cross-validation (or, for similar performance, the least data/computationally intensive). This process was repeated with the next set of hyperparameters. In a similar fashion, using 10-fold cross-validation, we evaluated the number of images used for training to ensure that approximately 60–70 images per data set yielded satisfactory results, with Dice scores above 0.90.

### U-Net cycleGAN configuration.

The U-Net cycleGAN was implemented in Python 3 with Tensorflow 2.0. The generator is made up of an encoder, a transformer, and a decoder. Based on Zhu et al. (20), the encoder consists of 3 convolutional layers with 64, 128, and 256 filters; kernel sizes 7, 3, and 3; and strides 1, 2, and 2. All layers use instance normalization, as well as ReLU activation. The transformer consists of 9 ResNet blocks (33), which are made up of 2 convolutional layers with instance normalization and ReLU activation for the first layer. The decoder is made up of 3 transposed convolutional layers. Before each decoder layer, the input is concatenated with the output of the corresponding layer in the encoder. The transposed convolutional layers have 128, 64, and 3 filters; kernel sizes 3, 3, and 7; and strides 2, 2, and 1. All layers use instance normalization, except for the first 2 layers, which are ReLU activated, and the last layer, which has a hyperbolic tangent (tanh) activation since its output is the generated image having pixel values between –1 and 1. The discriminator consists of 6 convolutional layers, with 64, 128, 256, 512, 512, and 1 filters; kernel size 4; and strides 2, 2, 2, 2, 1, and 1. Except for the first and last layer, all are instance normalized. And, except for the last layer, all use a leaky ReLU activation with an α slope of 0.2.

### CycleGAN training.

The model has been trained on 285 images from Controls 2 (Supplemental Table 1) and 180 images from Controls 1. For the validation, 46 images from Controls 2 and 44 from Controls 1 have been used. The images were resized to 256 × 256 pixels with 3 channels (RGB) using Gaussian pyramids. After their transformation, the images were upsampled to the original size of 1024 × 1024 pixels using Laplacian pyramids, as has been done in Engin et al. (34). The pyramids consist of layers calculated based on the original input. The network was trained for up to 200 epochs with a steady learning rate of 2 × 10^–4^ for the first 100 epochs and a linearly decaying learning rate that ends at 0 after 200 epochs. The cycle consistency loss and identity loss have been weighted with weights λ_cyc_ = 10 and λ_id_ = 5, respectively. The batch size was 1. The epoch with the lowest validation loss has been selected for transferring the images (epoch 83). The network was trained on an NVIDIA Quadro RTX 8000 48GB with TeslaLink.

Since bias between different data sets is not a new problem, a comparison between generative models and traditional approaches was necessary. Because the (initial) effort for generative models is higher, it should be shown that they lead to better results. As baseline methods, histogram equalization, color transfer based on a single reference image, and an adaptation of the mean colors to the reference have been tested. However, none of these methods showed a substantial improvement.

### Molecular podometrics.

Model-based stereology was applied (35) and allowed the estimation of podocyte number and podocyte density per glomerulus. Fiji imaging software (Max Planck Institute of Molecular Cell Biology and Genetics) was used to navigate the raw files. Podocytes were defined as DAPI^+^WT-1^+^DACH1^+^ cells. Glomerular cross-sectional areas were measured in order to estimate glomerular volumes and thereby define podocyte densities.

The morphometric signature combines the podometrics per glomerulus within each patient by calculating the minimum, maximum, mean, median, and variance of podocyte number, podocyte density, podocyte distance (distance to closest neighboring podocyte), podocyte nuclear area, and glomerular area across all glomeruli per subject.

### Data and materials availability.

The data sets generated and analyzed during the current study are available from the corresponding authors. The code is available via https://github.com/imsb-uke/podometric_u_net (Branch name: main, commit ID: a33afcc).

### Statistics.

All statistical analyses were performed using GraphPad Prism (v8.0.2) and Stata 13.1. Results are reported as median and IQR. Significance was evaluated using the unpaired Mann-Whitney *U* test when comparing 2 continuous variables. For comparison of 3 groups, Kruskal-Wallis test with Dunn’s multiple-comparisons test was used. Correlation analyses were performed using Spearman’s rank coefficients. A *P* value below 0.05 was considered to be statistically significant.

Classification of subjects into controls and ANCA-GN patients was performed in scikit-learn (36) using a logistic regression and leave-one-out cross-validation, where 1 subject was iteratively excluded from the training of the model and then used as a test set. The final results are a combination of all subjects’ results, each tested on a different model. Due to the nature of leave-one-out cross-validation without a completely unseen test set, no further optimization of parameters was possible. Each of the features in the morphometric signature was normalized by removing its mean and dividing by its SD before using it to train the logistic regression (excluding the test subject). To evaluate podometrics at the level of single glomeruli, individual glomeruli were clustered using ClustVis (37) based on podometrics via PCA by Pareto scaling to rows. Probabilistic PCA was used to calculate principal components.

### Study approval.

The ethics approval was obtained from the IRB of the RWTH Aachen University Medical Center, Germany (EK-016/17); the Ethik-Kommission der Ärztekammer Hamburg; and local ethics committee of the chamber of physicians in Hamburg (PV4806), all in accordance with the ethical principles stated by the Declaration of Helsinki.

## Author contributions

Study initiation was contributed by TBH, SB, and VGP. Conceptualization was contributed by MK, MZ, TBH, SB, and VGP. Methodology and analysis were contributed by MZ, MK, MNW, AKT, LG, CK, MH, JK, NW, FB, SW, TW, UP, CFK, EH, RK, SB, and VGP. Writing was contributed by MZ, MK, AKT, TBH, SB, and VGP. Supervision was contributed by TBH, SB, and VGP. Order of authors was decided based on timeline of authors’ contributions.

## Supplementary Material

Supplemental data

## Figures and Tables

**Table 1:**
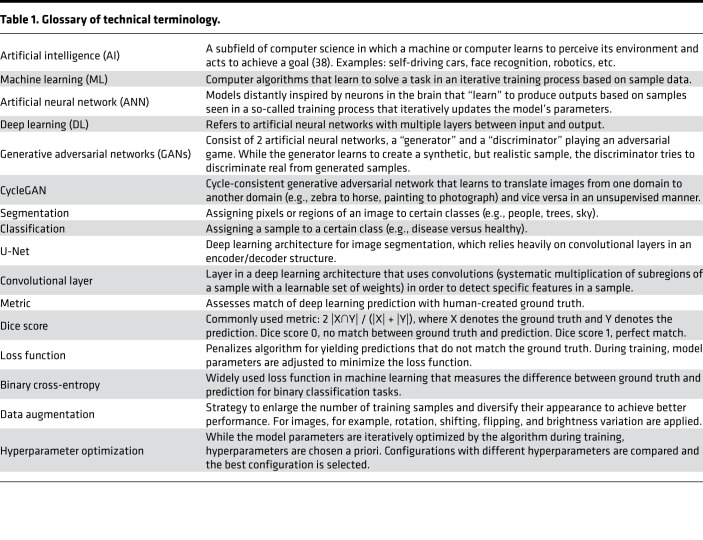
Glossary of technical terminology.

## Version 1 03/11/2021

In-Press Preview

## Version 2 04/08/2021

Electronic publication
