# Supplementary material for: Deep learning–based molecular morphometrics for kidney biopsies
Source: JCI Insight. 2021 Apr 8;6(7):e144779. doi: 10.1172/jci.insight.144779 (PMC8119189; doi:10.1172/jci.insight.144779)
Supplement: Supplemental data [file jciinsight-6-144779-s246.pdf]

Table S1. **Overview of all image datasets.** This table summarises all used subjects and images, divided by training, validation, test and unannotated sets in both controls and ANCA-GN patients. [$]controls 1 and 2 shared 16 subjects for a total of 48 analyzed controls. ANCA-GN: Anti-neutrophil cytoplasmic antibody-associated glomerulonephritis.

Dataset	Subset	# subjects	# images / glomeruli
Controls 1	Training	6	68
Controls 1	Validation	2	21
Controls 1	Test	2	20
Controls 1	Unannotated	16	168
Controls 1	All	26[$]	277
Controls 2	Training	5	64
Controls 2	Validation	2	20
Controls 2	Test	2	24
Controls 2	Unannotated	29	337
Controls 2	All	38[$]	445
ANCA-GN	Training	10	60
ANCA-GN	Validation	4	19
ANCA-GN	Test	3	21
ANCA-GN	Unannotated	45	273
ANCA-GN	All	62	373
		# subjects	# images / glomeruli
	**TOTAL**	110	1095

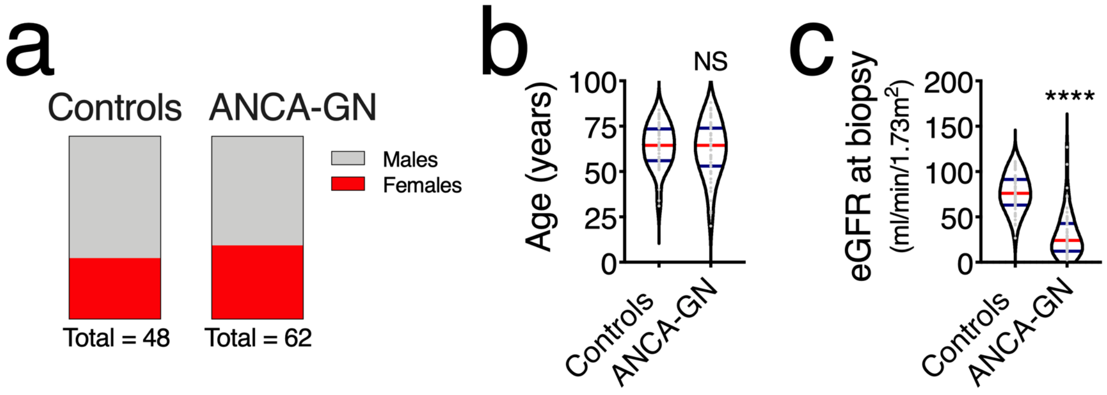

<u>**Figure S1.**</u> **Patient demographics.** (a) Similar rates of males and females between controls and ANCA-GN patients. (b) No age differences between controls and ANCA-GN patients. (c) Significant differences in estimated glomerular filtration rate (eGFR) between controls and ANCA-GN patients. In all panels n=48 for controls and n=62 for ANCA-GN patients; Mann Whitney's tests were performed. In violin plots, each grey dot represents one image, red lines represent medians and blue lines interquartile ranges. ANCA-GN: Anti-neutrophil cytoplasmic antibody-associated glomerulonephritis. ****P<0.0001 and NS: not statistically significant.

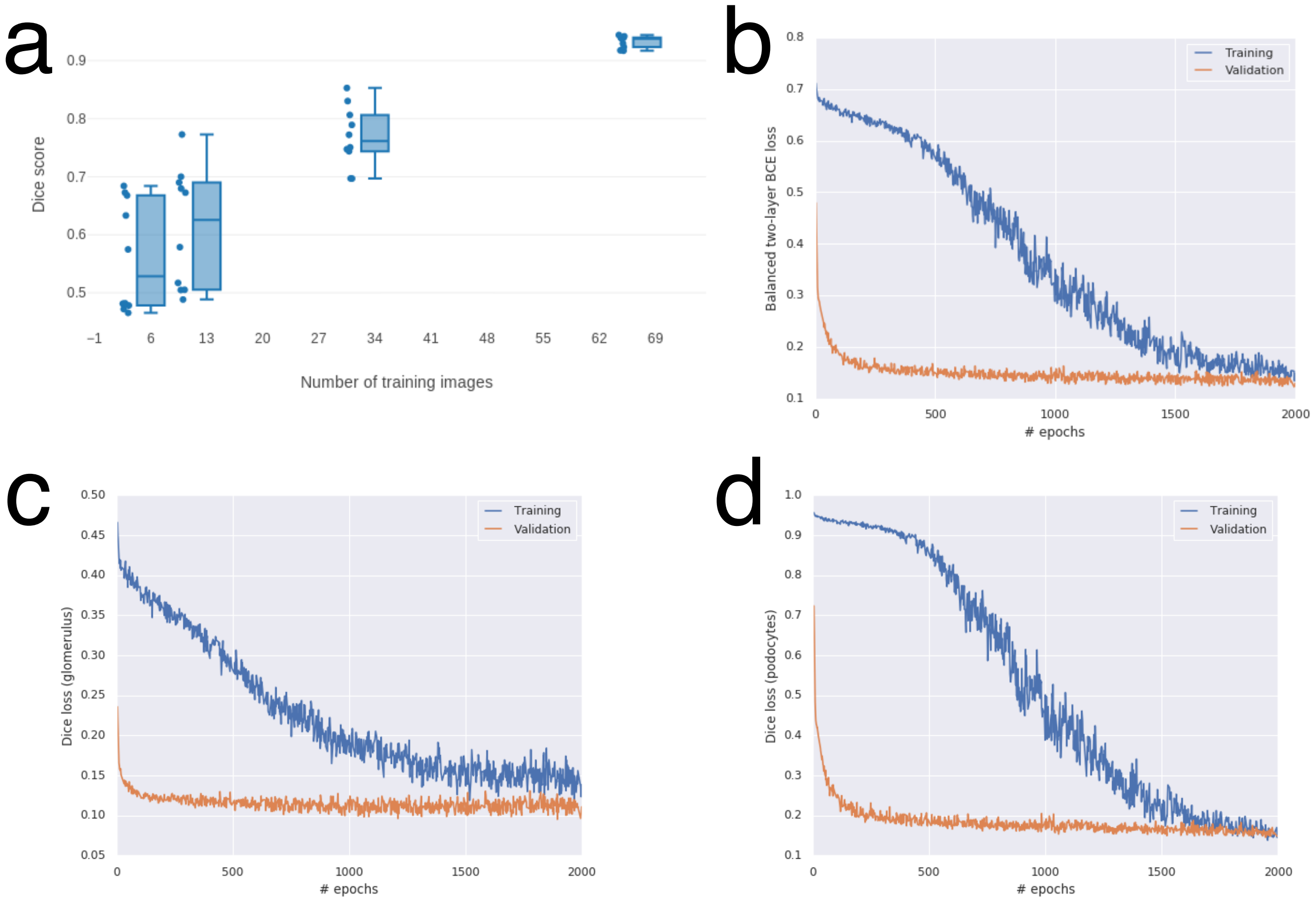

<u>**Figure S2.**</u> **Training of the dual segmentation U-Net.** (a) In a 10-fold cross validation, the number of training images was varied. In order to achieve Dice scores >0.90 on a dataset, approximately 65 images were required. The number of training images was scaled-up using the following steps: 6, 13, 34 or 68 images. The number of validation images was constant at n=21, the experiment was repeated 10 times. The training process with n=192 training and n=60 validation images was optimised using (b) the balanced 2-layer binary cross entropy (BCE) loss, weighting the individual BCE losses for the glomerulus and podocyte segmentation tasks. The performance was monitored using Dice losses for the (c) glomerulus and (d) podocyte segmentation (where the Dice loss = 1 - Dice score).

<u>Figure S3.</u> Comparison of single vs. dual output U-Nets. (a) Single and dual segmentation U-Nets provided comparable Dice scores, all over 0.90 for all segmentation tasks (n=21 images; Mann Whitney's tests were performed). (b) Visual representation of single and dual segmentation U-Nets' performance. GT: ground truth. Each blue dot represents a single image, red error bars represent medians and interquartile ranges. ****P<0.0001 and NS: not statistically significant.

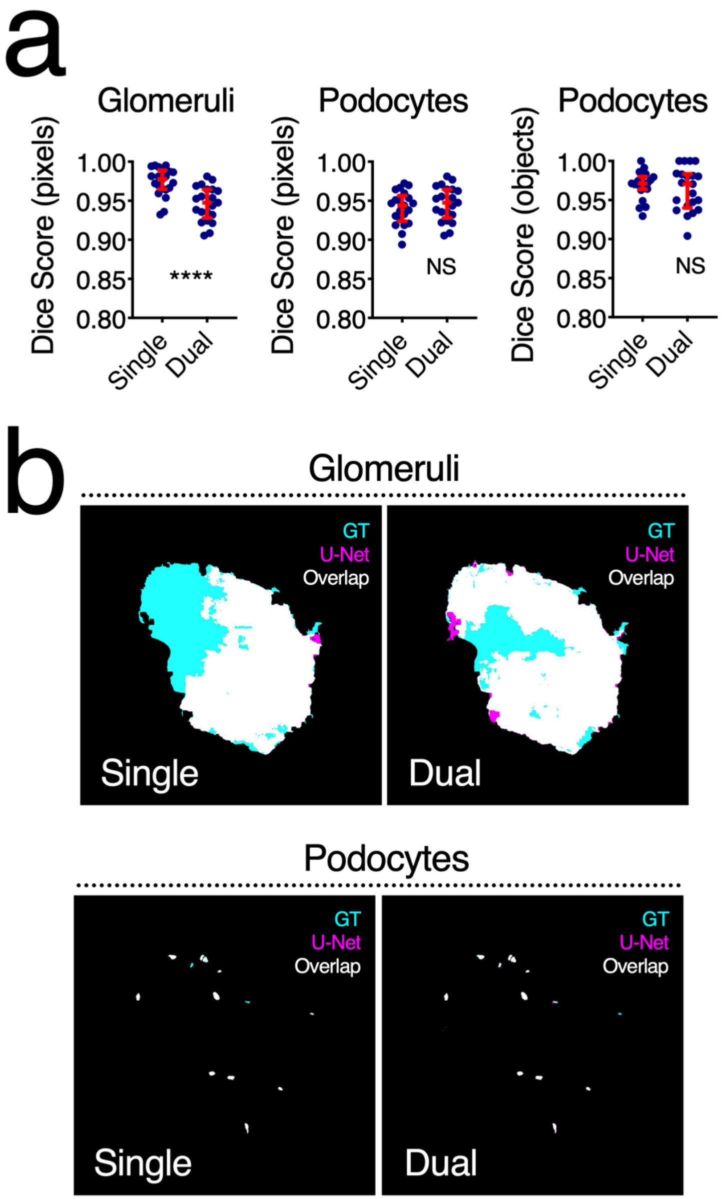

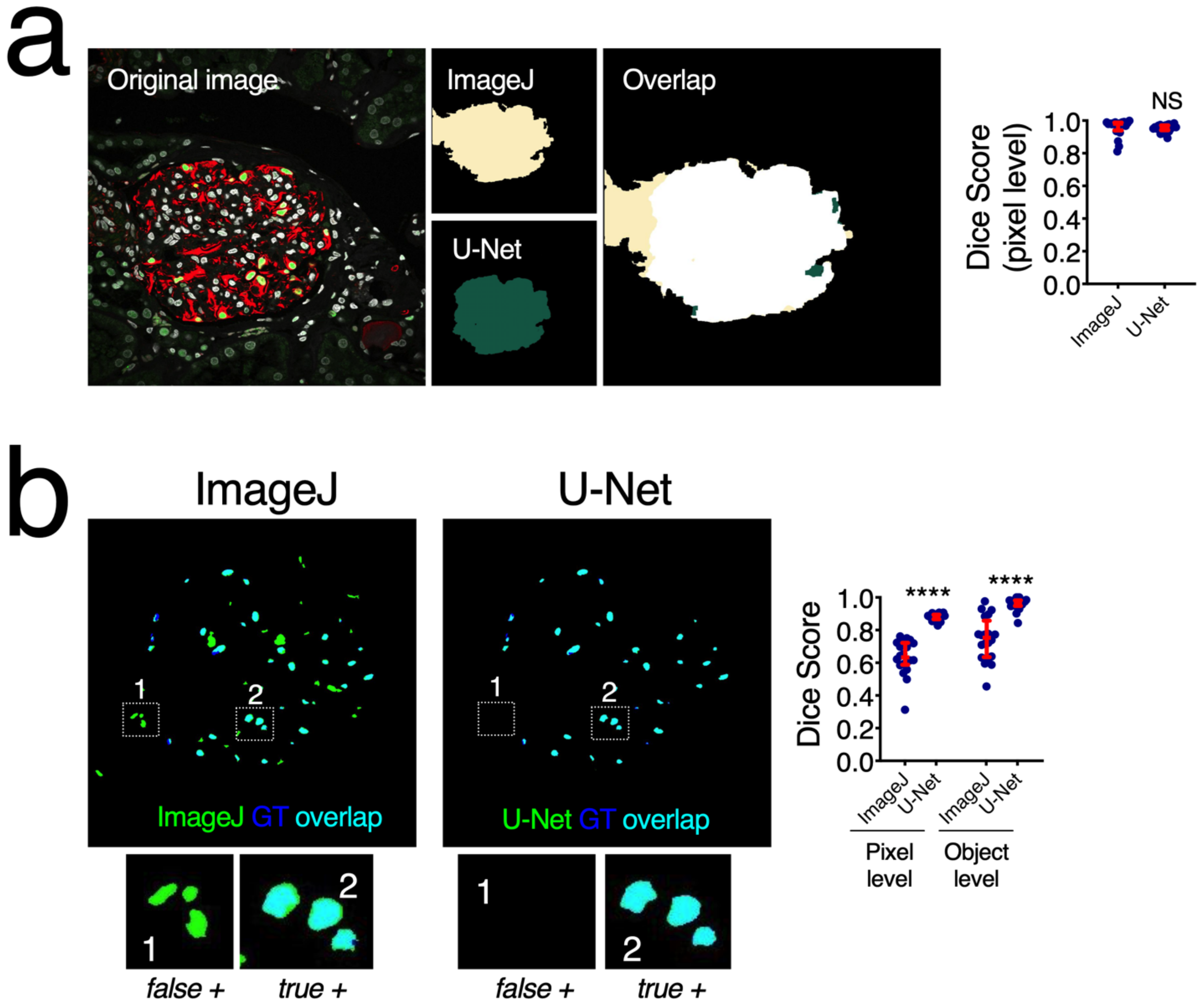

Figure S4. **Segmentation U-Net with dual outputs outperforms classical segmentation method.** (a) Glomerular segmentation was similar between the U-Net and an optimised ImageJ script. However, (b) our U-Net significantly outperformed the ImageJ script in podocyte segmentation both at a pixel and object level, reducing the presence of false positives. In both panels n=21 images were used; Mann Whitney's tests were performed. GT: ground truth, false +: false positives, true +: true positives. Each blue dot represents a single image, red error bars represent medians and interquartile ranges. ****P<0.0001 and NS: not statistically significant.

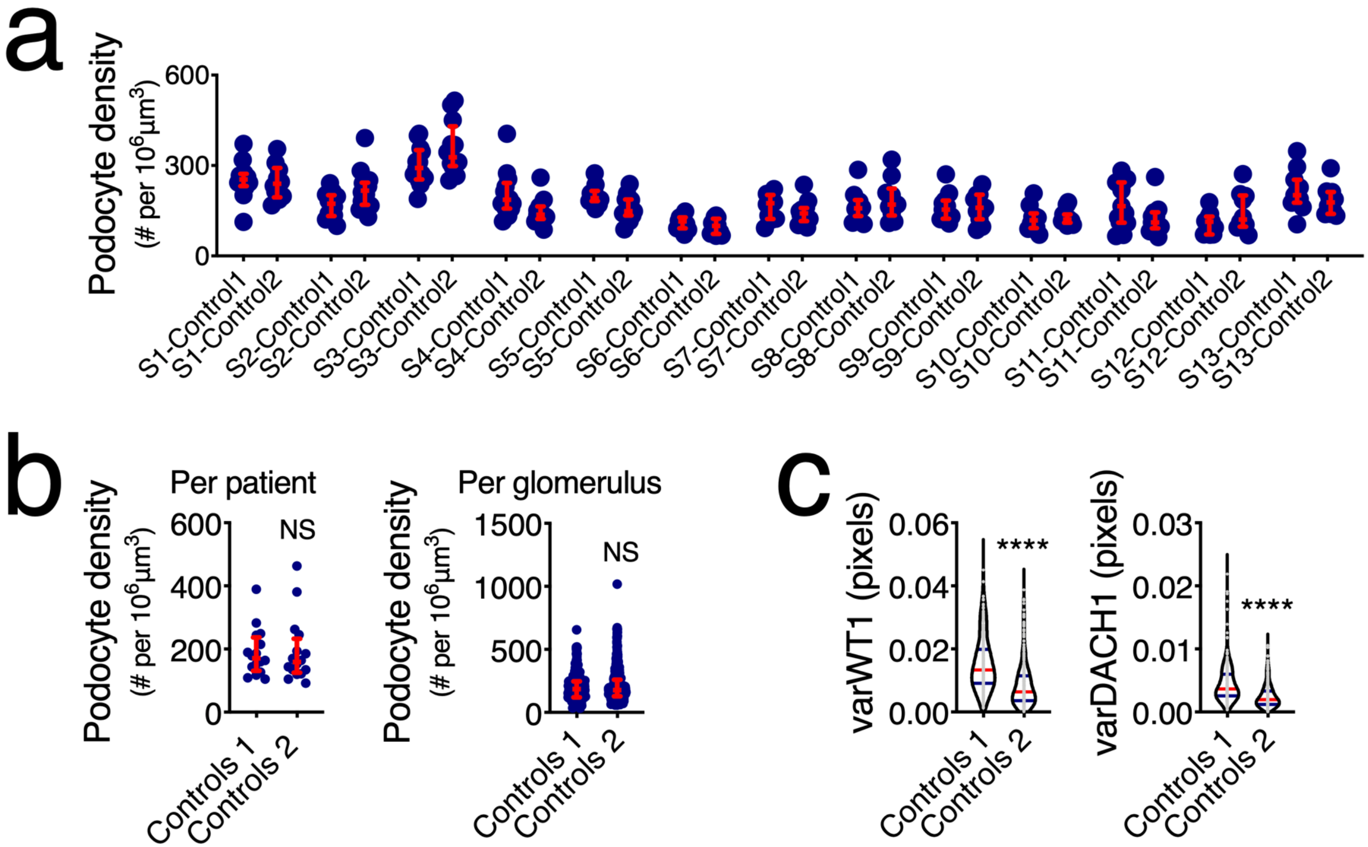

Figure S5. Potential batch effects in external datasets. (a) Direct comparisons of the podocyte density within the same subjects. Images were acquired in different sites by different operators using different microscopy systems and consist of 10 randomly sampled glomeruli (out of hundreds available per tissue). None of the comparisons showed statistical significance. (b) Group comparisons using medians per patient or every available glomerulus also showed no statistical differences. (c) Pixel-based analysis did show significant differences for variances in Wilms' Tumor 1 (WT1) and Dachshund Family Transcription Factor 1 (DACH1). In all panels n=21 glomeruli for controls 1 and n=20 glomeruli for controls 2; Mann Whitney's tests were performed. In dot plots, every blue dot represents one glomerulus in (a) and (b-right) and one patient in (b-left), and red error bars represent medians and interquartile ranges. In violin plots, each grey dot represents one image, red lines represent medians and blue lines interquartile ranges. ****$P<0.0001$ and NS: not statistically significant.

<u>Figure S6.</u> U-Net cycleGAN provides significant improvements in podocyte identification at both pixel and object level. (a) Training curves for the U-Net cycleGAN (the star indicates the best validation loss of the generator, which is the model chosen for evaluation), with n=180 training images from the internal dataset (A) and n=285 from the external dataset (B) and n=44 validation images from A and n=46 validation images from B. (b) ROC and precision-recall curves show the segmentation performance before and after transferring the images with the U-Net cycleGAN and in relation to the reference dataset (n=21 images for the internal dataset and n=20 images for the external dataset as before and after cycleGAN). (c) The best results are achieved with manual annotations and re-training of the segmentation U-Net. Using a U-Net cycleGAN leads to comparable Dice scores without the need to re-train the segmentation U-Net (n=20 images for the external dataset). TPR: true positive rate, FPR: false positive rate, AUC: area under the curve. In dot plots, every blue dot represents one image and red error bars represent medians and interquartile ranges.

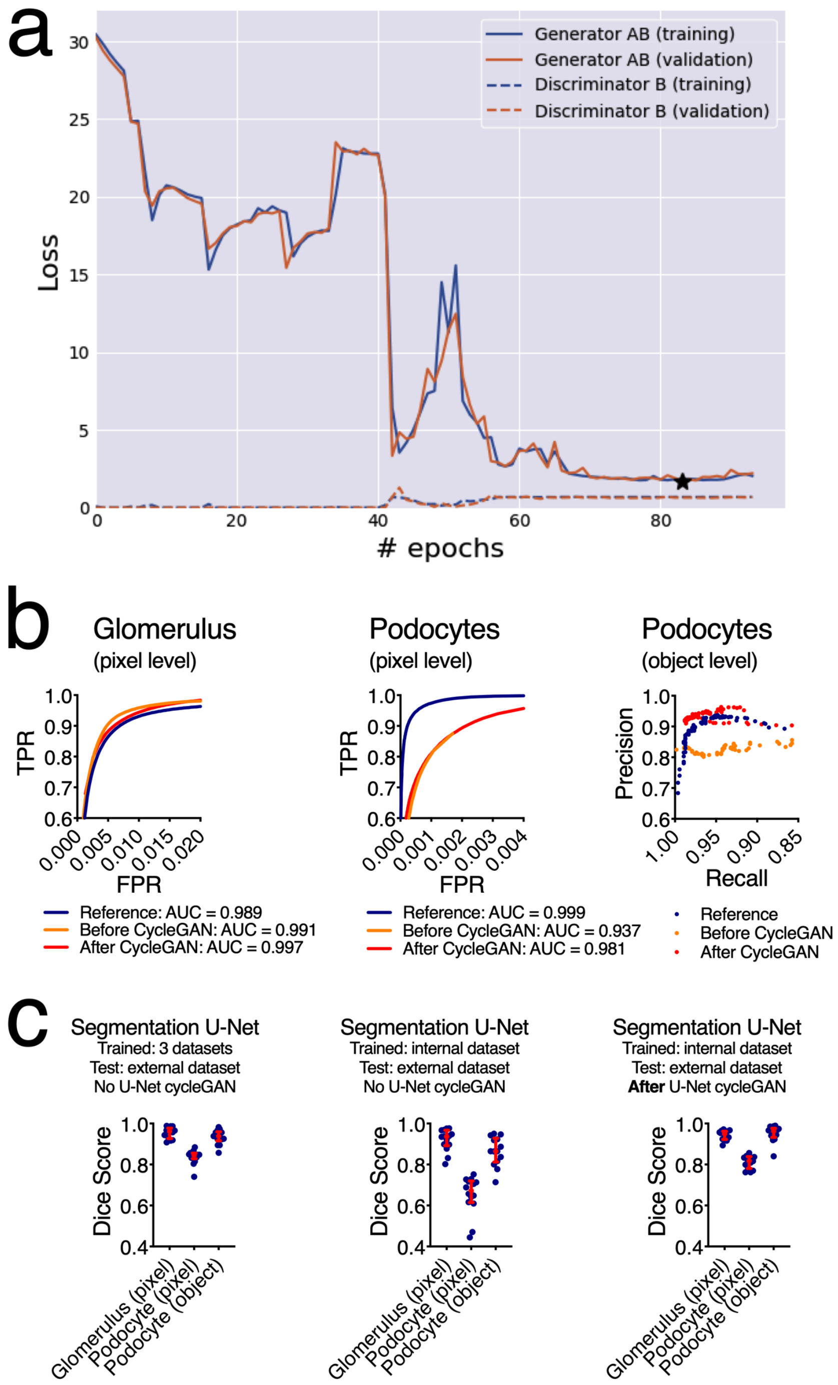

<u>Figure S7.</u> Biological validation of quantitative outputs from the segmentation U-Net. (a) 2D quantitative data derived from the manual segmentation (ground truth), including glomerular area, and podocyte count, showing morphometric changes in ANCA-GN patients. (b) 2D quantitative data derived from the segmentation U-Net, including glomerular area and podocyte count, showing identical results to ground truth. (c) Spearman rank correlation analyses of the podocyte count corrected for glomerular area confirmed strong agreement between ground truth and segmentation U-Net for both controls and ANCA-GN datasets. In all panels n=24 images from controls and n=21 images from ANCA-GN patients; Mann Whitney's tests were performed. In dot plots, every blue dot represents one image and red error bars represent medians and interquartile ranges. ANCA-GN: Anti-neutrophil cytoplasmic antibody-associated glomerulonephritis. ****P<0.0001, ***P<0.001, and **P<0.01.

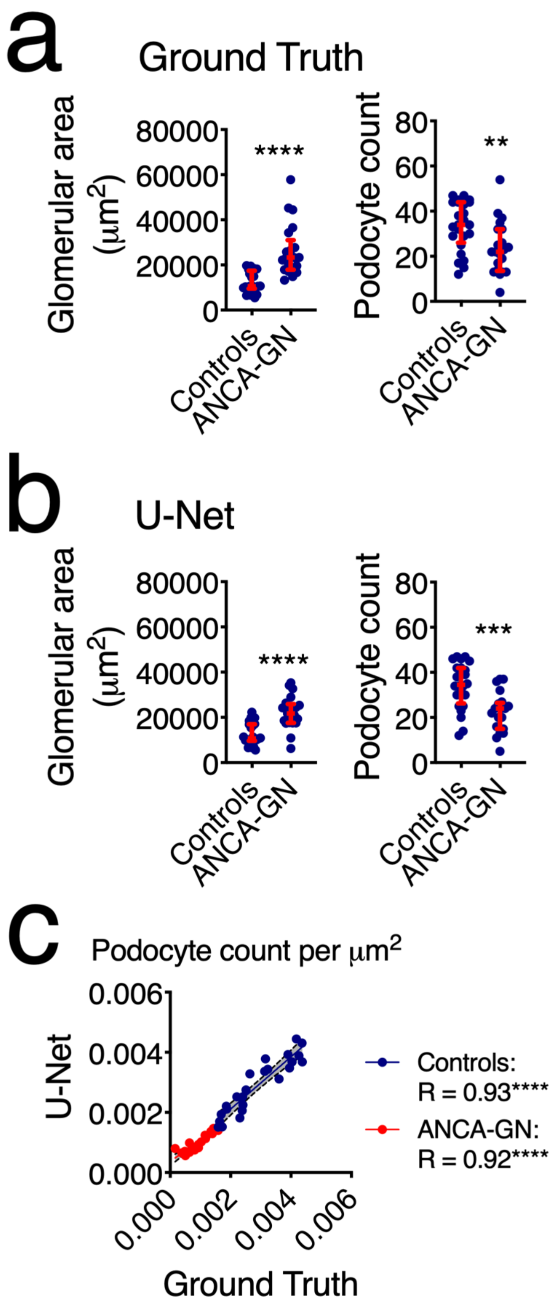

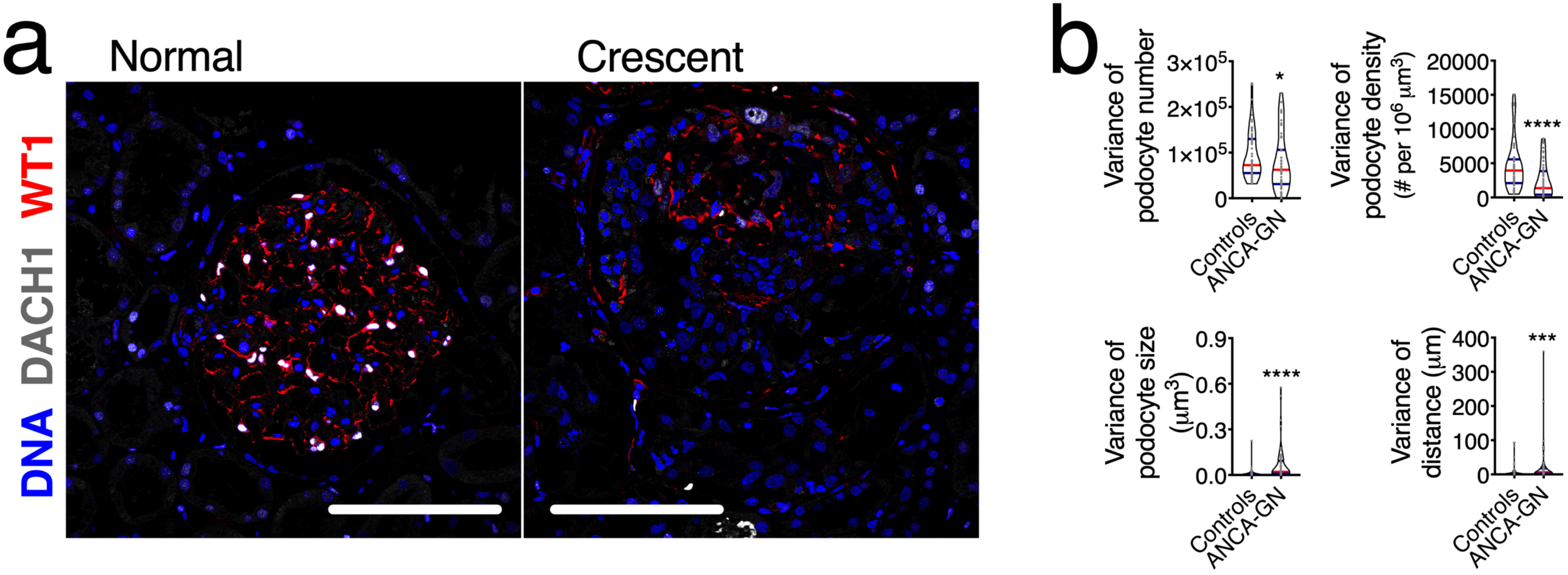

Figure S8. Variation within each biopsy. (a) Representative images of a normal glomerulus (left) and a pathological lesion (crescent, right). (b) Variance of podocyte number, density, size and distances to closest neighbours per subject, highlighting great variability within subjects that is directly affected by the development of kidney disease. In (b) n=48 controls and n=62 ANCA-GN patients; Mann Whitney's tests were performed. Scale bars represent 150µm. ANCA-GN: Anti-neutrophil cytoplasmic antibody-associated glomerulonephritis, DACH1: Dachshund Family Transcription Factor 1, WT1: Wilms' Tumor 1. ****P<0.0001, ***P<0.001, and *P<0.05.

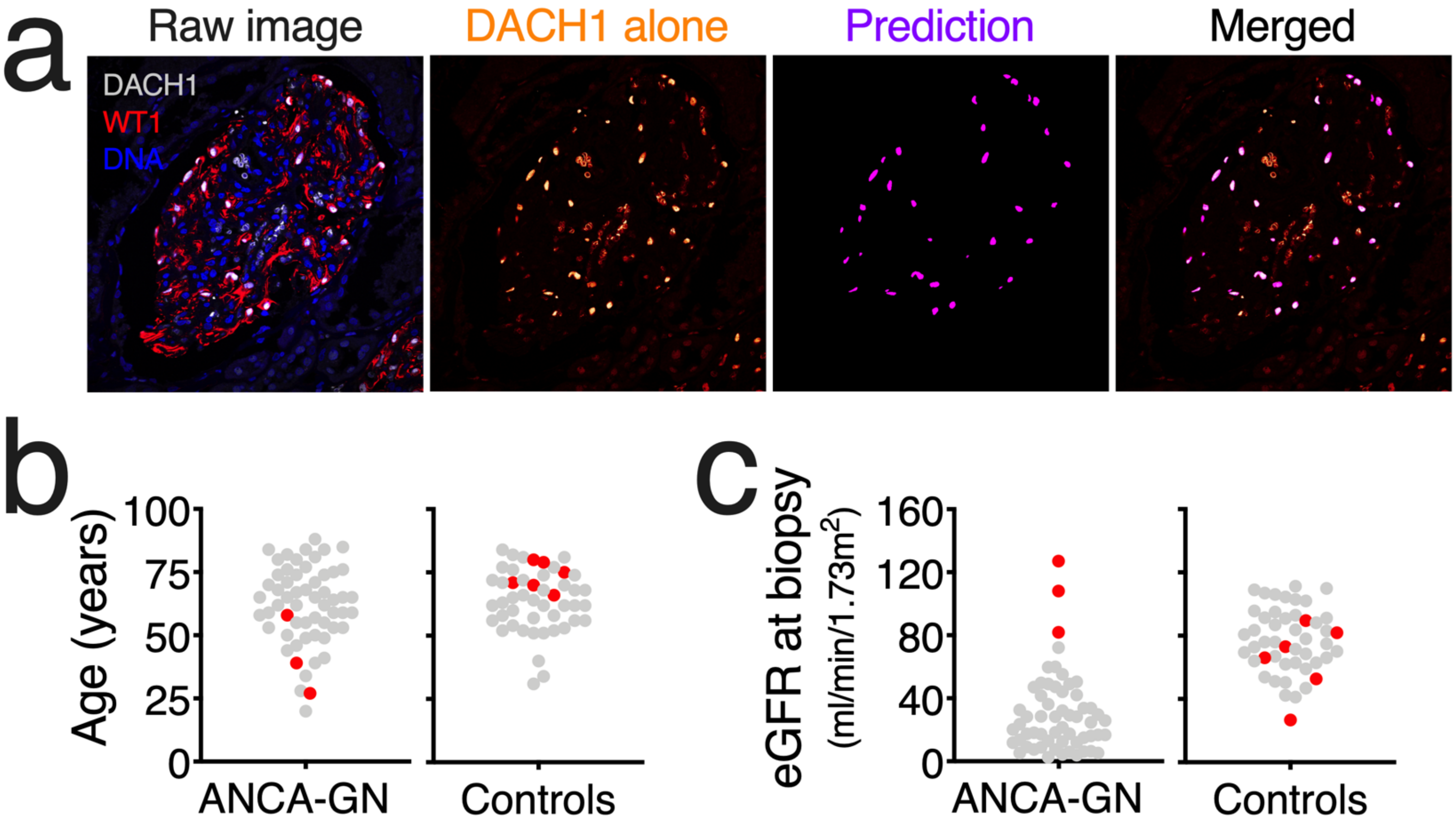

Figure S9. Potential causes of subject misclassification. (a) Representative images of a glomerulus from a misclassified subject, showing high prediction accuracy despite the unspecific signal of Dachshund Family Transcription Factor 1 (DACH1). (b) Identification of misclassified subjects (red) in the context of age distributions for ANCA-GN and controls. (c) Identification of misclassified subjects (red) in the context of estimated glomerular filtration rate (eGFR) distributions for ANCA-GN and controls. In (b) and (c) n=48 controls and n=62 ANCA-GN patients. ANCA-GN: Anti-neutrophil cytoplasmic antibody-associated glomerulonephritis, WT1: Wilms' Tumor 1.
